# AgMYB5, an MYB transcription factor from celery, enhanced β-carotene synthesis and promoted drought tolerance in transgenic *Arabidopsis*

**DOI:** 10.1186/s12870-023-04157-3

**Published:** 2023-03-21

**Authors:** Miao Sun, Qin-Yi Xu, Zhi-Peng Zhu, Pei-Zhuo Liu, Jian-Xiang Yu, Yao-Xian Guo, Shu Tang, Zhi-Fang Yu, Ai-Sheng Xiong

**Affiliations:** 1 College of Marine and Biological Engineering, Yancheng Teachers University, Yancheng, 224002 Jiangsu China; 2 State Key Laboratory of Crop Genetics and Germplasm Enhancement, College of Horticulture, Nanjing Agricultural University, Nanjing, 210095 Jiangsu China; 3 College of Food Science and Technology, Nanjing Agricultural University, Jiangsu, 210095 China

**Keywords:** AgMYB5, β-carotene, Drought tolerance, Abscisic acid, Y2H, Celery

## Abstract

**Background:**

Water shortage caused by global warming seriously affects the yield and quality of vegetable crops. β-carotene, the lipid-soluble natural product with important pharmacological value, is abundant in celery. Transcription factor MYB family extensively disperses in plants and plays regulatory roles in carotenoid metabolism and water scarcity response.

**Results:**

Here, the *AgMYB5* gene encoding 196 amino acids was amplified from celery cv. ‘Jinnanshiqin’. In celery, the expression of *AgMYB5* exhibited transactivation activity, tissue specificity, and drought-condition responsiveness. Further analysis proved that ectopic expression of *AgMYB5* increased β-carotene content and promoted drought tolerance in transgenic *Arabidopsis thaliana*. Moreover, *AgMYB5* expression promoted β-carotene biosynthesis by triggering the expression of *AtCRTISO* and *AtLCYB*, which in turn increased antioxidant enzyme activities, and led to the decreased contents of H_2_O_2_ and MDA, and the inhibition of O_2_^−^ generation. Meanwhile, β-carotene accumulation promoted endogenous ABA biosynthesis of transgenic *Arabidopsis*, which resulted in ABA-induced stomatal closing and delayed water loss. In addition, ectopic expression of *AgMYB5* increased expression levels of *AtERD1*, *AtP5CS1*, *AtRD22*, and* AtRD29.*

**Conclusions:**

The findings indicated that *AgMYB5* up-regulated β-carotene biosynthesis and drought tolerance of *Arabidopsis*.

**Supplementary Information:**

The online version contains supplementary material available at 10.1186/s12870-023-04157-3.

## Introduction

Celery (*Apium graveolens* L.) is one of the world's most important vegetable crops in the Apiaceae family [1]. Traditionally, celery leaf blades and petioles have long been appreciated for their nutritional and pharmacological properties [2, 3]. In contrast to other vegetables, celery is rich in a range of nutrients, including carotenoids, ascorbic acid, apigenin, and flavonoids [4, 5]. At the same time, celery contains a vast array of secondary metabolites with properties of anti-oxidative damage, anti-inflammation, and anti-diabetes. Due to global warming and irregular rainfall, both celery production and quality are negatively affected by drought stress [6].

Transcription factors (TFs) have attracted attention because of their vital role as upstream regulators of structural genes. MYBs [7], NACs [8], bHLHs [9], and WRKYs [10] have been proven the potential capacity of regulating plant drought tolerance. Moreover, by interacting directly with abscisic acid (ABA), TFs played positive regulatory roles in the development and drought stress of plants. In addition, TFs from *Vitis vinifera* [11], *Oryza sativa* [12], and *Zea mays* [13], were found to be the negative regulators of plant drought tolerance.

In plants, MYBs are composed of one of the most important families of TFs [14]. As well as regulating organ development, MYBs widely participate in tissue formation and is important for response mechanisms to multiple environmental stresses [15]. In recent years, MYBs from *Triticum aestivum* [16], *Sesamum indicum* [17], and *Lycopersicon esculentum* [18], have been proven the function of regulating drought tolerance. There is evidence that MYBs respond to drought stress by coordinating the stomatal opening and closing [19], influencing flower development [20], regulating phytohormone signal transduction [21], and affecting cuticular wax accumulation [22].

In addition, MYBs act as key regulators of promoting and repressing carotenoid metabolism in plants [23]. As the major source of retinol in humans, carotenoids have the efficacies of anti-oxidative, anti-cancer, immunomodulatory, and delaying senescence. However, the human body is unable to synthesize carotenoids, therefore, we must obtain them through plant-based foods. β-carotene, belonging to the branch of carotenoids, contributes to the treatment of eye diseases, cardiovascular disorders, and neoplasms [24]. In plants, β-carotene is biosynthesized in plastids through the metabolism of terpenoids and polyketides, and mediated by various enzymes, including phytoene desaturase (PDS), ζ-carotene desaturase (ZDS), carotenoid isomerase (CRTISO), and lycopene β-cyclase (LCYB) [25]. Recent studies suggested that in *Vitis vinifera* [26], *Capsicum annuum* [27], and *Ulva prolifera* [28], overexpression of *MYBs* promoted or repressed β-carotene accumulation. However, the regulatory role of MYBs in abiotic resistance and carotenoid accumulation in celery still lacks a thorough understanding.

To obtain new insights into the biological function of the MYB family in celery, based on our published celery genome information [2, 4] and the transcriptomic data of celery under drought stress [29, 30], the gene encoding the transcription factor AgMYB5 was amplified from celery cultivar ‘Jinnanshiqin’ and heterologously expressed in *A. thaliana*. Physiological traits of wild type (WT) and transgenic *Arabidopsis*, including the contents of total carotenoid, β-carotene, malondialdehyde (MDA), hydrogen peroxide (H_2_O_2_), and superoxide anion-radicals (O_2_^−^), as well as the activities of superoxide dismutase (SOD), peroxide (POD), catalase (CAT), were detected and analyzed. Moreover, water loss rate, ABA content, stomatal aperture, and relative expression profiles of drought-stress-responsive genes were also measured and analyzed. Our work demonstrated that *AgMYB5* played potentially vital roles in β-carotene accumulation and drought tolerance of plants.

## Materials and methods

### Plant material, culture conditions, and abiotic stress treatment

The seeds of celery (*A. graveolens* L., cv. ‘Jinnanshiqin’) and *Arabidopsis thaliana* (‘Columbia-0’) were kindly provided by the State Key Laboratory of Crop Genetics and Germplasm Enhancement, Nanjing Agricultural University (32°04′N, 118°85′E). Celery was incubated in the plant cultivation laboratory of Yancheng Teachers University (33°38’N, 120°20’E). The culturing temperature and day length was set at 24 °C for 16 h of day-time and 18 °C for 8 h of night-time. To gain insights into the expression pattern of *AgMYB5* in celery under abiotic stress, two-month-old healthy celery plants were cultured under four kinds of abiotic stress conditions, heat (38 °C treatment), cold (-7 °C treatment), salt (200 mM NaCl treatment), and drought (200 g/L PEG 6000 treatment) stress. Celery samples were collected at 6 h, 12 h, and 24 h after stress treatments, and quickly frozen in liquid nitrogen.

*A. thaliana* (ecotype ‘Columbia-0’) was used for *AgMYB5* transformation. In the artificial climate chamber, climatic conditions were 24 °C, 70% relative humidity, and long photoperiod of 15 / 9 (light/dark). Here, WT and *AgMYB5* transgenic *Arabidopsis* seeds were sterilized with 75% ethanol and 10% sodium hypochlorite, germinated on Murashige and Skoog (MS) solid medium, transferred to soil in pots (nine seedlings per pot) and grown under normal conditions for three weeks. One week later, all *Arabidopsis* plants were treated with drought stress (200 g/L PEG 6000 treatment) for 7 d. Then, *A. thaliana* growth conditions were observed and analyzed, while its physiological and molecular indexes were assayed.

### *AgMYB5* amplification and bioinformatic analysis

Total RNA of celery and *A. thaliana* was extracted using CTAB isolating buffer (Catalog No. R21004, purchased from Shanghai Yuanye Biotechnology Co., Ltd), and cDNA was synthesized with *EasyScript*® Reverse Transcriptase (Catalog No. AE101-02, purchased from Beijing Transgene Biotechnology Co., Ltd). In PCR amplifications with *AgMYB5*-specific primers, cDNA was used as a template. In addition, the upstream and downstream primers were designed with DNAMAN 9.0 and synthesized by Nanjing GenScript Biotechnology Co., Ltd. Nanjing GenScript Biotechnology Co., Ltd. also undertook the task of PCR product sequencing.

ExPASy Translate Tool (https://web.expasy.org/translate/) was used to translate nucleic acid sequences of *AgMYB5* into amino acid sequences. ExPASy-Protparam tool (https://web.expasy.org/protparam/) was used to calculate the physiochemical properties of AgMYB5. NCBI BLAST tool (https://blast.ncbi.nlm.nih.gov/Blast.cgi) was used for the search of *MYB5* in the other plant species. The secondary and tertiary structure prediction of AgMYB5 was conducted by SOPMA (https://npsa-prabi.ibcp.fr/) and Swiss-Model (http://swissmodel.Expasy.org/), respectively. Multiple amino acid sequence alignment of MYB5s was performed using DNAMAN 9.0, and the *NJ* phylogenetic tree was built by MEGA 7.0.

### *A. thaliana* transformation and assay for drought tolerance evaluation

The ORF product of *AgMYB5* was obtained with primers (Forward primer: 5’-TTTACAATTACCATGGGGAGAAGCCCGTGTTGTTCCA-3’, reverse primer: 5’-GTTTCGACTGATTGCTTCTGCCT-3’) and digested with *Bam* HI and *Sac* I. After that, *AgMYB5* ORF was introduced into *Agrobacterium* GV3101 by the pCAMBIA-1301 vector, and the floral dip method [31] was utilized to transform *A. thaliana*. Transgenic *A. thaliana* were screened using hygromycin (50 mg/L, w/v) and continuously cultured to obtain homozygous T3 transgenic *A. thaliana* lines.

The copy number of exogenous *AgMYB5* in transgenic *Arabidopsis* was measured with a previous method [32]. Briefly, genomic DNA was extracted from the leaves of *AgMYB5* transgenic *Arabidopsis* lines and diluted into five-fold. Through qRT-PCR, the *C*q values of *At4*-*HPPD* and *AgMYB5* were detected and the standard curves of *At4*-*HPPD* and *AgMYB5* were conducted. Then, the copy number of *AgMYB5* was calculated according to the formula [33].

In vitro evaluation for drought tolerance of *A. thaliana* was conducted according to the method [34]. *A. thaliana* seeds were germinated on solid MS medium supplement with PEG 6000 (w/v, 200 g/L) for 14 d. Afterward, the growth characteristics of these *A. thaliana* seedlings were observed and analyzed. Besides, in vivo evaluation was determined by the method [35]. Briefly, four-week-old WT and *AgMYB5* transgenic *A. thaliana* seedlings in pots were treated with PEG 6000 solution for 7 d. Then, the stomatal aperture [36] of *Arabidopsis* abaxial epidermal peels and the water loss rate [37] of *Arabidopsis* samples were determined.

### Determination of total carotenoid and β-carotene

The total carotenoid content was measured according to the acetone-hexane method [38]. Briefly, 2.0 g of *A. thaliana* samples were frozen and ground in liquid nitrogen. The powder was extracted with an acetone-hexane mixture and cold stood for 10 min. After decanting and running the solution through a 0.45 mm filter, the extract’s absorbance was measured reflecting the total carotenoid content.β-carotene content was determined by the method [39]. The powder, described above, was incubated at 50 °C for 1 h. After filtration with 0.45 μm, 20 μL of the extract was injected into Vanquish Flex system for ultra-high performance liquid chromatography (UPLC) analysis. Acetonitrile and methanol (1:9, v/v) were applied as the eluants with 0.8 mL/min of the flowing rate. After that, the effluent was monitored simultaneously at 450 nm by a diode array detector.

### Measurement of ABA, MDA, H_2_O_2_, O_2_^−^, and antioxidant enzymes

An assay of MDA content, SOD and POD activities was performed based on the measurement [40]. The generation rate of O_2_^−^ was measured following the previous method [41]. ABA content was measured with a Plant ABA ELISA kit (Catalog No. H251-1). CAT activity was assayed with a Plant CAT ELISA Kit (Catalog No. A007-1). H_2_O_2_ content was measured based on the Plant H_2_O_2_ ELISA Kit (Catalog No. A064-1). In addition, these ELISA kits were acquired from Nanjing Jiancheng Biological Engineering Co., Ltd.

### Quantitative real-time PCR (qRT-PCR)

In this experiment, *A. graveolens* gene expression levels were quantified and normalized to *AgGAPDH* [42], while *A. thaliana* gene expression in WT and transgenic lines were normalized with *AtActin3*. *AgMYB5*, ABA synthesis-related genes (*AtABA1*, *AtNCED6*, *AtABA2*, *AtAAO3*), β-carotene synthesis-related genes (*AtPDS3*, *AtZDS*, *AtCRTISO*, *AtLCYB*), and drought response-related genes (*AtERD1*, *AtP5CS1*, *AtRD22*, *AtRD29*), whose relative expression levels were determined by qRT-PCR (Primer information in Table 1). The qRT-PCR reaction was conducted by TransStart® Top Green qPCR SuperMix (Catalog number: AQ131-01, purchased from Beijing Transgene Biotechnology Co., Ltd.). Besides, the *C*_T_ method [43] was used to calculate gene expression relative to the references.

**Table 1:** Nucleotide sequences and characteristics of primers

Gene	Encoding enzyme	Forward primer (5’ -3’)	Reverse primer (5’ -3’)	Product length (bp)
*AgMYB5*	v-myb avian myeloblastosis viral	CAAGCTCTTGGGGAACAGGT	AGGTTGGGGAGCCAGGATAA	292
*AgGAPDH*	glyceraldehyde-3-phosphate dehydrogenase	CTCGCCCCGACATCTCTTTC	TCACTGGTCCGTGACTTTGG	202
*AtActin3*	β-actin	CCTTACAGAAGCACCAGCTCA	GTCCAGCAAGGTCTAAACGGA	241
*AtABA1*	zeaxanthin epoxidase	GCATTTCACGAGGAACCAGC	CCCATATTTGGCTGCATCGC	230
*AtNCED6*	nine-cis-epoxycarotenoid dioxygenase	CAGGTAACTTCGCTCCGGTT	AGTGTACCGGCAGCTGTAAC	215
*AtABA2*	short-chain dehydrogenase/reductase	CACCGTGCCCTGATATTCGT	GTTCACACGTATCCCGTGCT	263
*AtAAO3*	abscisic aldehyde oxidase	GTTATGCAGAGACCGACCCC	CTCTCGGATGTCGTGCTACC	245
*AtPDS3*	phytoene desaturase	CACTGTCGAAGGAGACGCTT	ATCCATTCCTCTGCTGGTGC	297
*AtZDS*	zeta-carotene desaturase	GTGCTCCTCGTCCTGCTAAG	GTGCTGGTAGGACATCTGGG	242
*AtCRTISO*	carotenoid isomerase	ACGTTCAAGGAGGTGGGAAC	CCCGATGAGCACACATCACT	217
*AtLCYB*	lycopene epsilon cyclase	CGAGGAGACATGTTTGGCCT	CTGAATAGCCTGTTGCGGGA	218
*AtERD*	early response to dehydration protein	AAGGAAAAGAGCCACCCTCG	AGTTTCTGCCAGGTAGCGTC	214
*AtP5CS*	delta-pyrroline-5-carboxylate synthetase	CGTTGGCATGCACAGTTGAA	CCAACCTCTGCACCTAGTCC	211
*AtRD22*	responsive to dehydration 22	TCAGCTCCACGACGATCCTA	GAACCAGCTTCCACCGAGAA	204
*AtRD29*	desiccation-responsive 29	GAGTCACAGTTGACACGTCCT	TCCATGTTTAGTGAGACTGTTCT	243

### Transcriptional activity assay by Y2H

The ORF that encodes *AgMYB5* was inserted into the pGBKT7 plasmid and shuttled into the Y2H vector (Catalog number: PG1029, purchased from Wuhan Protein Interaction Biotechnology Co., Ltd.). Meanwhile, Y2H vectors with pCL1 and empty pGBKT7 plasmids were set as positive and negative controls, respectively. Over two days, the solid medium was placed in a stationary position upside down to initiate cultivation at 30 °C. In the YPDA liquid medium, positive clones were cultivated overnight to turbidity. After centrifuging at 5000 rpm for 5 min, the sediment was diluted with ddH_2_O and inoculated on SD/-His/-Ade solid medium (X-gal).

### Statistical analysis

To determine whether there were any significant differences in physiological indexes and gene expression profiles between WT and *AgMYB5* transgenic *Arabidopsis* lines, the data were statistically analyzed with point t-test, one-way and two-way ANOVA and graphed with GraphPad 7.0. A significant difference was defined as ^*^P < 0.05 and ^**^P < 0.01. All plant lines were compared by Tukey’s multiple range test (P < 0.05). In addition, each experiment was repeated three times and included three biological replicates.

## Results

### Bioinformatic analysis of AgMYB5

**Fig. 1:**
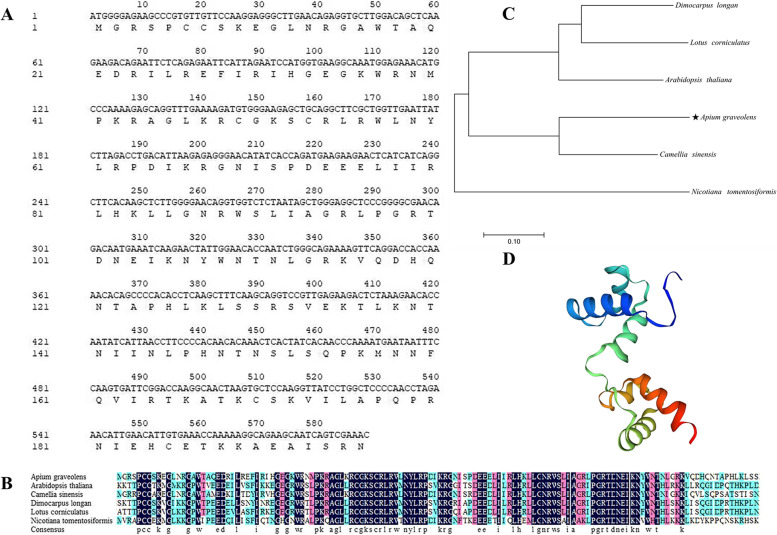
Sequence analysis of *AgMYB5* gene. **A** Nucleotide sequence and predicted amino acid sequence of *AgMYB5.* **B** Multiple amino acid sequence alignment of AgMYB5 (OP272489), AtMYB5 (NP_187963.1), CsMYB5 (QSV39846.1), DlMYB5 (QRV61369.1), LcMYB5 (QXT50540.1), and NtMYB5 (XP_009601374.1). **C** The *NJ* phylogenetic tree of AgMYB5, NtMYB5, SlMYB5, OsMYB5, and MeMYB5. **D** Protein tertiary structure prediction of AgMYB5

Bioinformatic analysis showed that the *AgMYB5* gene (NCBI accession No. OP272489) contained 588 nucleotide base pairs and 196 amino acids in the ORF (Fig. 1A), with the predicted molecular weight and theoretical *pI* value of 22.65 kDa and10.44, respectively. Multiple amino acid sequence alignment revealed a reasonable degree of sequence similarity between AgMYB5 and other MYB5s from *A. thaliana* (NP_187963.1), *Camellia sinensis* (QSV39846.1), *Dimocarpus longan* (QRV61369.1), *Lotus corniculatus* (QXT50540.1), and *Nicotiana tomentosiformis* (XP_009601374.1) (Fig. 1B). Among them, AgMYB5 shared the highest similarity with CsMYB5 (32.58%). The *NJ* phylogenetic tree constructed by MEGA 7.0 also revealed close evolutionary relationships between AgMYB5 and CsMYB5 (Fig. 1C). Besides, protein structure prediction suggested that AgMYB5 protein was composed of the alpha helix (31.63%), extended strand (6.63%), beta-turn (6.12%), and random coil (55.61%) (Fig. 1D).

### Transcriptional activity assay and function prediction of *AgMYB5*

**Fig. 2:**
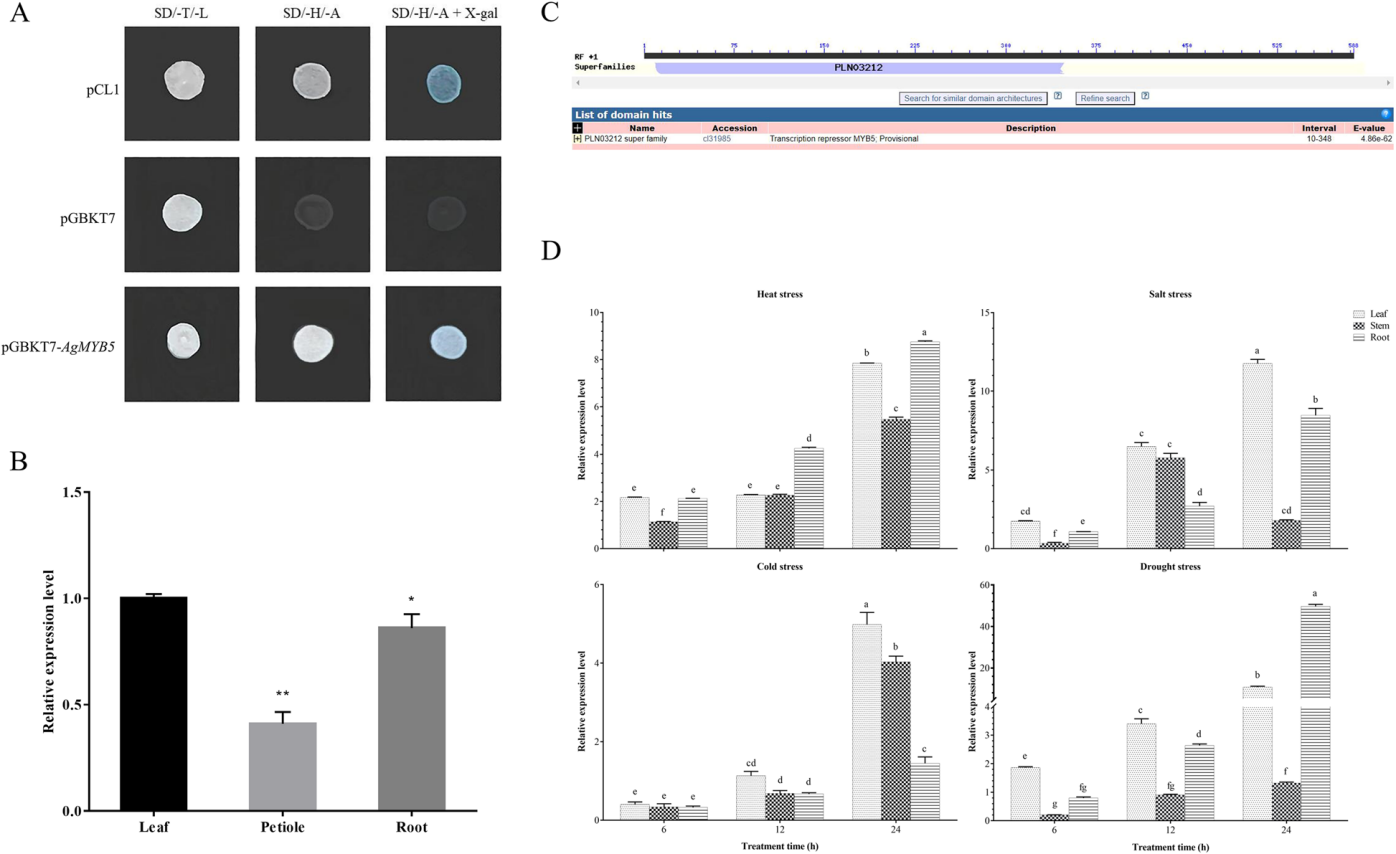
Bioinformatic analysis and expression characteristics of *AgMYB5*. **A** Verification of transcript activation ability by *Y2H*. **B** The relative expression level of *AgMYB5* in different tissues of ‘Jinnanshiqin’. The asterisk indicates a statistically significant difference (^*^, *P* < 0.05; ^**^, *P* < 0.01). **C** Conserved domain analysis of *AgMYB5*. **D** The relative expression level of *AgMYB5* in different tissues of ‘Jinnanshiqin’ under heat (38 °C), cold (-7 °C), salt (200 mM NaCl), and drought (200 g/L PEG 6000) conditions. Significant differences are indicated by different lettered columns (*P* < 0.05)

The vector of pGBKT7-*AgMYB5* was constructed to verify the transcriptional activity of the *AgMYB5* gene. As shown in Fig. 2A, on SD/-His/-Ade solid medium, positive control of pCL1 grew normally and appeared blue when incubated in X-gal. As the negative control, pGBKT7 plasmid was found to grow normally on SD/-Trp/-Leu solid medium but was inviable on SD/-His/-Ade solid medium. Moreover, the pGBKT7-*AgMYB5* plasmid was found to grow normally on SD/-Trp/-Leu and SD/-His/-Ade solid mediums and appeared blue on SD/-His/-Ade medium containing X-gal. These results demonstrated that the AgMYB5 protein could activate the transcription of reporter genes *His3* and *Laz*, suggesting that *AgMYB5* was capable of transcriptional activation.

Using qRT-PCR, a comparison of *AgMYB5* expression profiles among different tissues was presented in Fig. 2B. *AgMYB5* exhibited differentiated expression patterns in various tissues of celery (cv. Jinnanshiqin). Compared with that of the petiole (*P* < 0.01) and root (*P* = 0.03), the highest expression of *AgMYB5* was obtained in the leaf. Besides, conserved domain analysis revealed that the *AgMYB5* gene contained an MYB5 domain (Fig. 2C), which was related to abiotic stress response [44]. Therefore, the qRT-PCR analysis demonstrated that *AgMYB5* exhibited specific expression patterns under various abiotic stresses (Fig. 2D). Notably, with drought treatment for 24 h, compared with that of 6 h treatment, the relative expression level of *AgMYB5* increased by 4.97 folds in the leaf, 5.56 folds in the petiole, and 58.51 folds in the root, respectively. The above results demonstrated that *AgMYB5* was highly responsive to various abiotic stresses, especially drought stress.

### Promoted β-carotene biosynthesis of *AgMYB5 *transgenic *Arabidopsis*

**Fig. 3:**
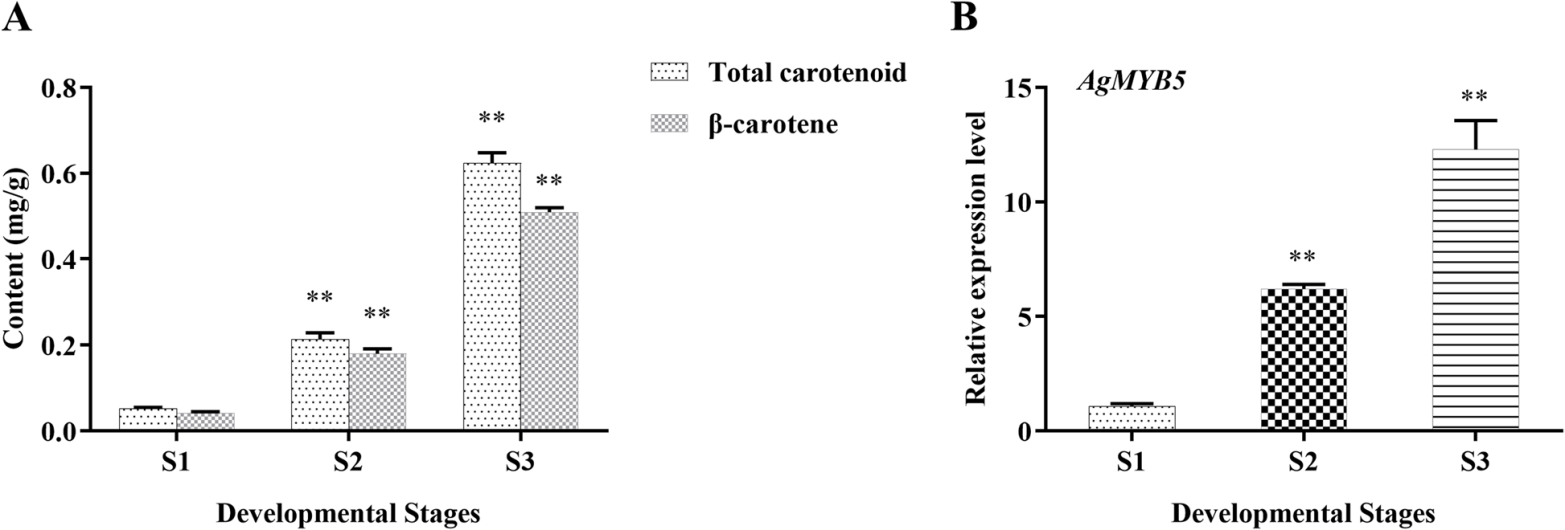
Total carotenoid and β-carotene accumulation and *AgMYB5 expression* in different developmental stages of ‘Jinnanshiqin’. Three developmental stages of celery included S1 (35 d post-planting), S2 (50 d post-planting), and S3 (65 d post-planting). **A** The total carotenoid and β-carotene contents and **(B)** relative expression level of *AgMYB5* during different developmental stages. The asterisk indicates a statistically significant difference (^*^, *P* < 0.05; ^**^, *P* < 0.01)

The contents of total carotenoid and β-carotene of celery (cv. Jinnanshiqin) samples at three developmental stages, including stage 1 (S1, 35 d post-planting), stage 2 (S2, 50 d post-planting), and stage 3 (S3, 65 d post-planting), were assayed. At the same time, the *AgMYB5* expression level was also determined. The results showed that total carotenoid content increased from 0.052 mg/g at S1 to 0.625 mg/g at S3, and β-carotene content increased from 0.42 mg/g at S1 to 0.51 mg/g at S3 (Fig. 3A). Meanwhile, the expression profiles of *AgMYB5* increased by 4.17 folds (S2) and 10.33 folds (S3) as compared to that of S1 (Fig. 3B). These results implied that *AgMYB5* contributed to β-carotene accumulation.

**Fig. 4:**
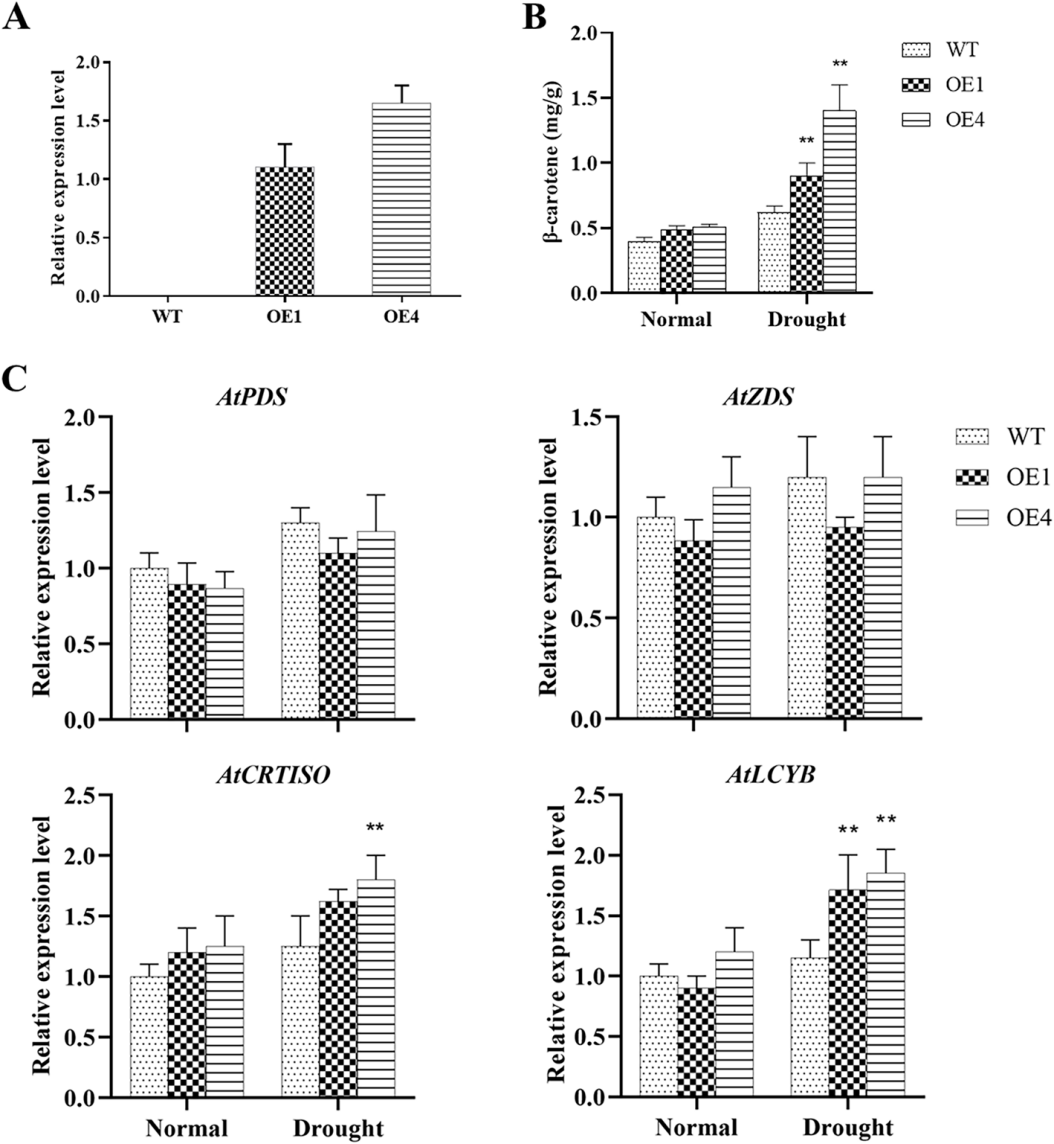
Validation of the positive *AgMYB5* transgenic *Arabidopsis* lines. **A** The transcript levels of *AgMYB5* in transgenic *Arabidopsis* lines (OE1 and OE4). **B** β-carotene content and **(C)** relative expression levels of β-carotene biosynthesis-related genes in WT and transgenic *Arabidopsis* lines (OE1 and OE4). The asterisk indicates a statistically significant difference (^*^, *P* < 0.05; ^**^, *P* < 0.01)

To further verify the role of *AgMYB5* in β-carotene biosynthesis, we obtained homozygous *AgMYB5* transgenic *Arabidopsis* (OE1 and OE4) through self-pollination. The copy number of exogenous *AgMYB5* was about one in OE1 and OE4 (Supplementary Figure S1), and qRT-PCR results showed that the expression level of *AgMYB5* was slightly higher in the independent transgenic line of OE4 (Fig. 4A). The β-carotene content assay showed that under normal conditions, OE1 and OE4 accumulated slightly more β-carotene than that of WT, and drought stress led to significant accumulation of β-carotene in transgenic *Arabidopsis* lines (Fig. 4B), especially between the group OE4 *vs* WT (*P* < 0.001). Furthermore, the qRT-PCR analysis demonstrated that no significant changes in the expression of *AtPDS* and *AtZDS* were detected under normal and drought conditions, while under drought stress, *AtCRTISO* (*P* = 0.009) and *AtLCYB* (*P* = 0.001) in OE4 showed significantly higher expression levels in comparison with that in WT (Fig. 4C). These results indicated that ectopic expression of *AgMYB5* significantly enhanced β-carotene biosynthesis of transgenic *A. thaliana* by regulating the expression of *AtCRTISO* and *AtLCYB*.

### Enhanced drought tolerance of *AgMYB5 *transgenic *Arabidopsis*

**Fig. 5:**
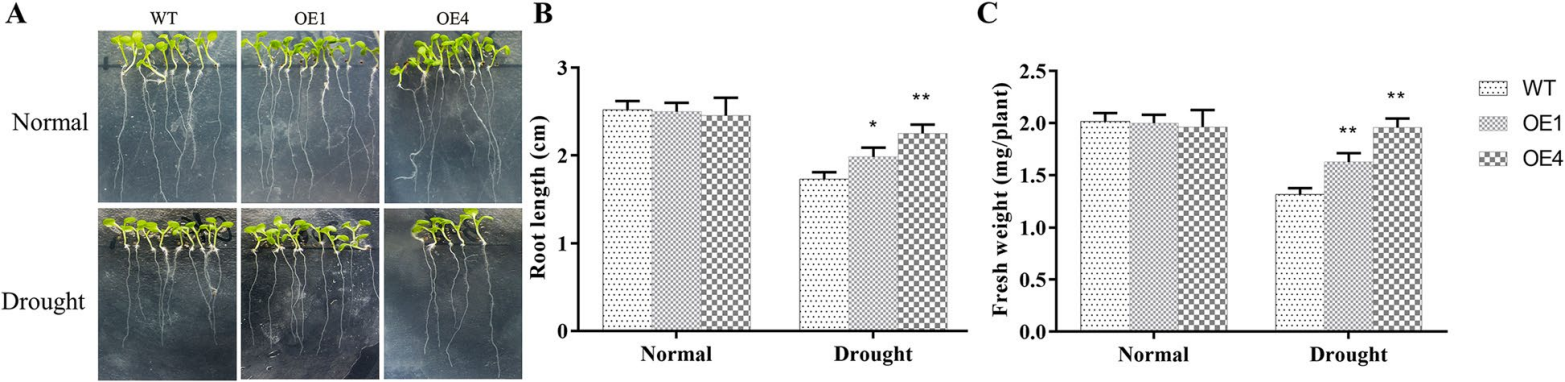
Drought tolerance of WT and transgenic *Arabidopsis* lines (OE1 and OE4) germinated on MS-agar plates. **A** The Phenotype of WT and transgenic lines under normal and drought conditions. **B** Average Root length and **(C)** fresh weight of WT and transgenic lines under normal and drought conditions. The asterisk indicates a statistically significant difference (^*^, *P* < 0.05; ^**^, *P* < 0.01)

To investigate the regulatory roles of *AgMYB5* under drought conditions, *Arabidopsis* seeds were germinated on MS solid medium and MS medium containing PEG 6000 (200 g/L). Under normal conditions, a similar growth phenotype was observed in WT and *AgMYB5* transgenic *Arabidopsis* lines. However, under drought stress, OE1 and OE4 lines presented a better growth situation (Fig. 5A). The data showed that under drought stress, compared to WT, the average root length (Fig. 5B) and fresh weight (Fig. 5C) of OE4 increased by 30.06% (*P* = 0.004) and 48.85% (*P* < 0.001), respectively.

**Fig. 6:**
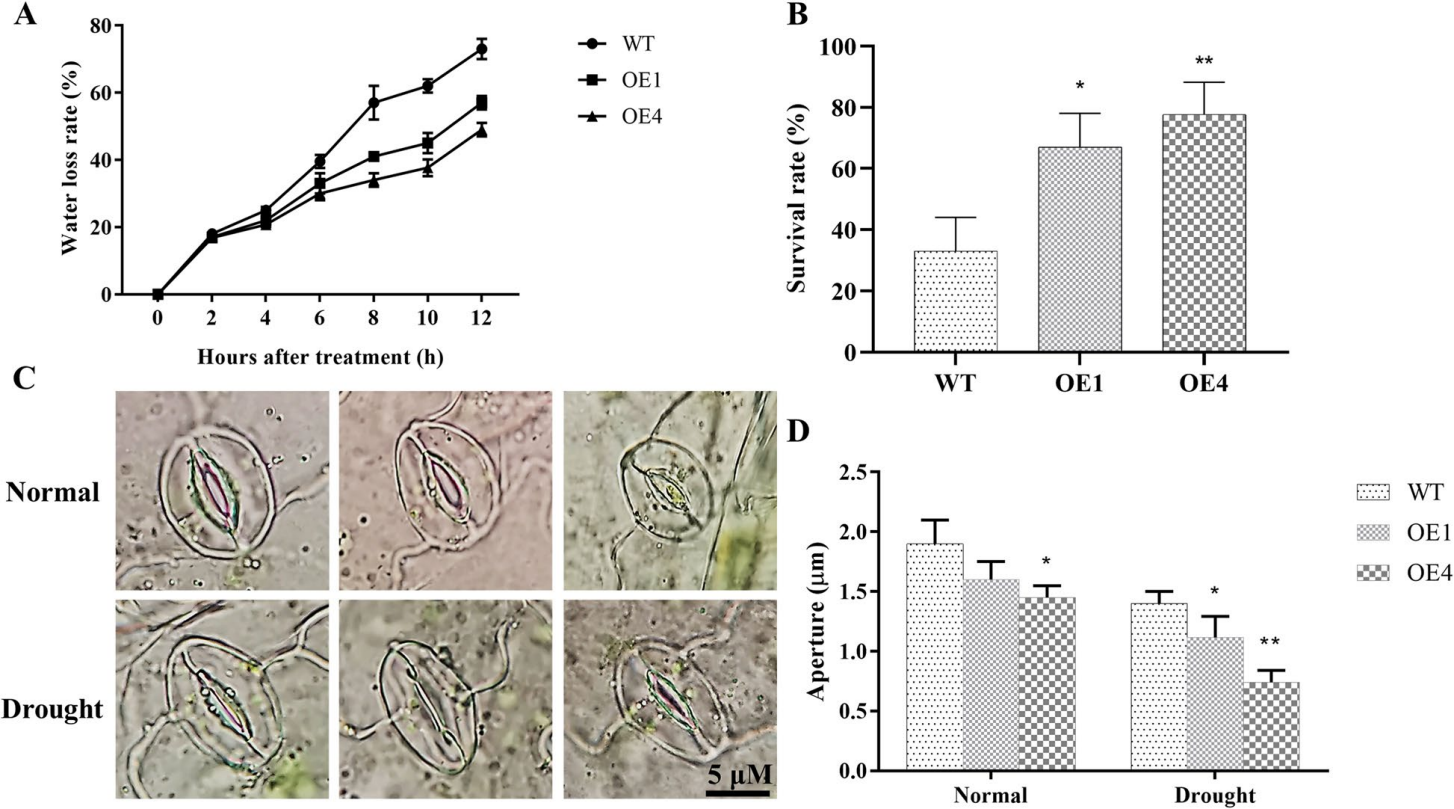
Drought tolerance of WT and transgenic *Arabidopsis* lines planted in soil. **A** The water loss rate (percent water loss per hour) and **(B)** the survival rate of WT and transgenic lines (OE1 and OE4). **C** The extent of stomatal closure and **(D)** stomata aperture of WT and transgenic lines under normal and drought conditions. The asterisk indicates a statistically significant difference (^*^, *P* < 0.05; ^**^, *P* < 0.01)

In vitro drought tolerance evaluation showed that four-week-old WT and *AgMYB5* transgenic *A. thaliana* seedlings in pots (nine plants with the same age per pot) displayed similar developmental patterns. Meanwhile, under drought stress, all WT seedlings wilted and lost water significantly, while *AgMYB5* transgenic lines displayed mild wilting. With the extension of the PEG 6000 treatment, the water loss rate of transgenic line OE4 was the lowest, while that of WT was the highest (Fig. 6A). After drought treatment, transgenic line OE1 and OE4 had a significantly higher survival rate, which increased by 2.03-fold (OE1) and 2.66-fold (OE4) than WT, respectively (Fig. 6B). Because stomatal closure may be the first response of plants to drought stress, as well as observing the state of stomata, we also measured the stomata aperture of WT and transgenic lines by ImageJ software (Fig. 6C). Under normal conditions, the stomata aperture of transgenic line OE4 was slightly lower as compared to that of WT and transgenic line OE1. Under drought stress, all *Arabidopsis* plants exhibited decreased stomata aperture in varying degrees. Among these, the stomata aperture of OE4 was the lowest (0.74 μm), followed by OE1 (1.12 μm) and then WT (1.44 μm) (Fig. 6D). These results demonstrated that *AgMYB5* expression significantly enhanced drought tolerance of transgenic *A. thaliana* by inducing stomatal closure.

### Promoted ABA biosynthesis of *AgMYB5 *transgenic *Arabidopsis*

**Fig. 7:**
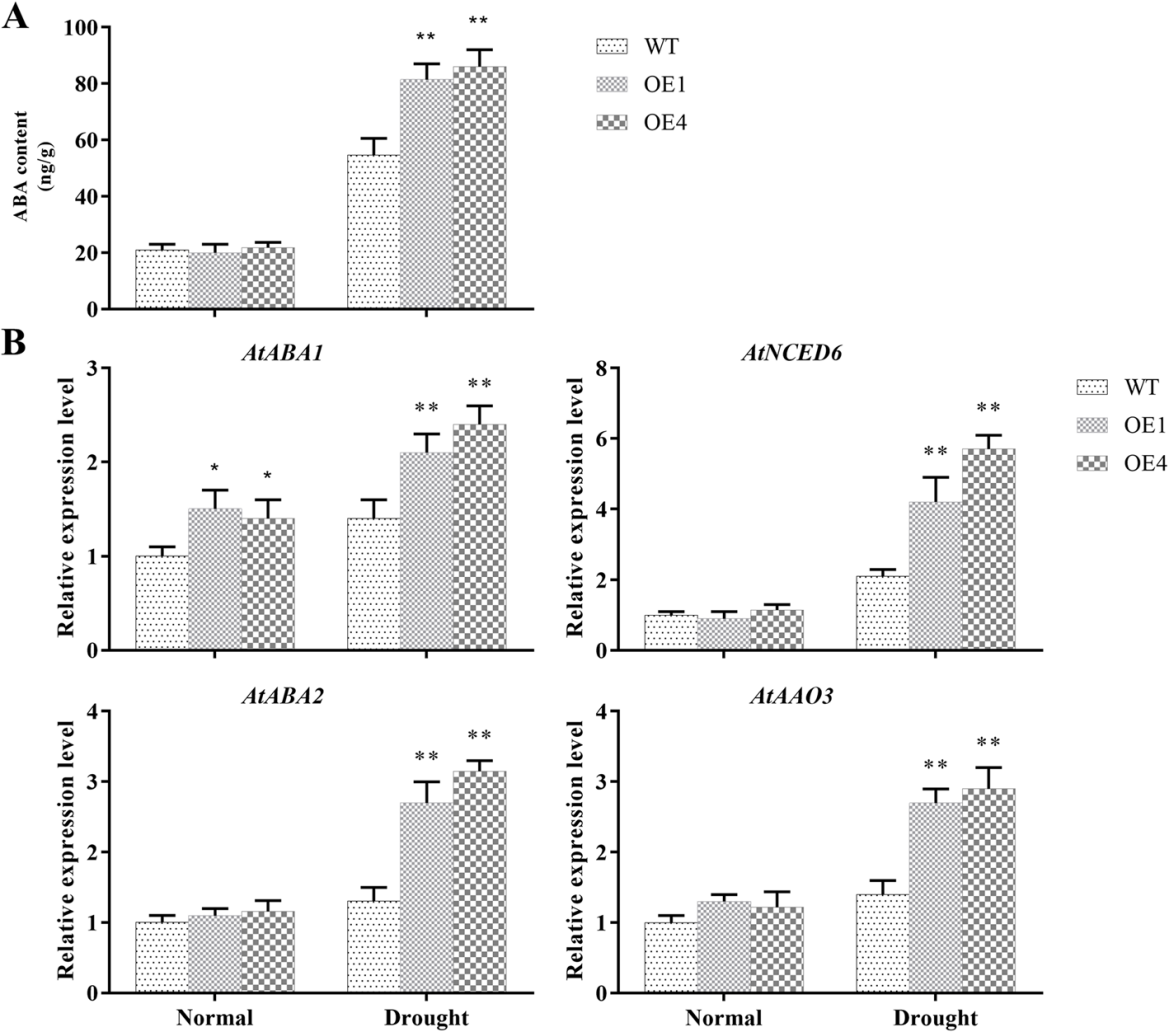
Effects of *AgMYB5* expression on endogenous ABA biosynthesis pathway. **A** Endogenous ABA contents and **(B**) relative expression levels of ABA synthesis-related genes (*AtABA1*, *AtNCED6*, *AtABA2*, and *AtAAO3*) of WT and transgenic lines under normal and drought conditions. The asterisk indicates a statistically significant difference (^*^, *P* < 0.05; ^**^, *P* < 0.01)

Since ABA is derived from β-carotene and can cause stomatal closure, a measurement of endogenous ABA content in *Arabidopsis* was conducted. Under normal conditions, no significant differences in endogenous ABA content were assayable between WT and *AgMYB5* transgenic *A. thaliana* lines (OE1 and OE4). However, under drought stress, endogenous ABA content of OE1 and OE4 increased by 3.05 folds and 2.91 folds, respectively, whereas that of WT increased by only 1.61 folds (Fig. 7A). The qRT-PCR analysis further examined the expression of ABA synthesis-related genes, including *AtABA1*, *AtNCED6*, *AtABA2*, and *AtAAO3*. The data exhibited that in transgenic lines under drought conditions, four genes encoding ABA biosynthesis were up-regulated. Particularly *AtNCED6*, encoding the rate-limiting enzyme in ABA biosynthesis, whose expression in transgenic line OE4 was 2.71 times greater than that in WT (Fig. 7B). These findings indicated that the expression of *AgMYB5* in transgenic *A. thaliana* activated the endogenous ABA synthesis pathway.

### Protected ROS scavenging system of *AgMYB5 *transgenic *Arabidopsis*

**Fig. 8:**
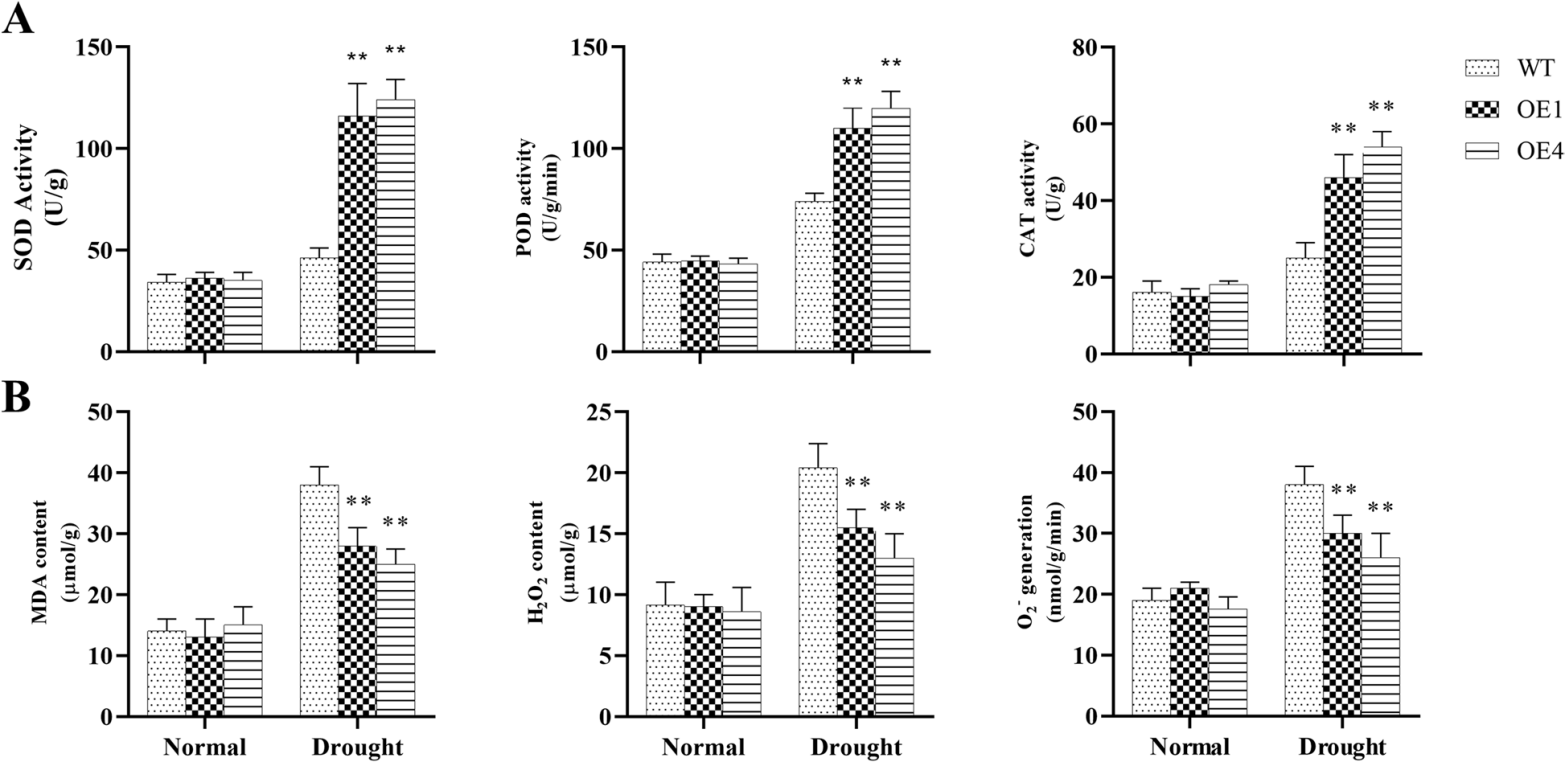
Antioxidant capacity of WT and transgenic lines under normal and drought conditions. **A** Activities of SOD, POD, and CAT enzyme and **(B)** contents of MDA, H2O2, and O_2_^−^ generation of WT and transgenic lines (OE1 and OE4). The asterisk indicates a statistically significant difference (^*^, *P* < 0.05; ^**^, *P* < 0.01)

Under drought stress, plants respond to excessive accumulation of ROS by promoting antioxidant enzyme activities and protecting the ROS scavenging system [45]. Thus, we analyzed the antioxidative enzymatic activities and ROS contents of *A. thaliana* under normal and drought conditions. Compared to WT, *AgMYB5* transgenic *A. thaliana* showed increased SOD, POD, and CAT activities by 152.17% (OE1) and 169.57% (OE4), 48.65% (OE1) and 62.16% (OE4), and 84.02% (OE1) and 116.11% (OE4), respectively (Fig. 8A). At the same time, comparing *AgMYB5* transgenic lines with WT, we observed decreases in MDA content, H_2_O_2_ content, and O_2_^−^ generation of 26.32% (OE1) and 34.21% (OE4), 24.02% (OE1) and 36.27% (OE4), and 21.05% (OE1) and 31.58% (OE4) (Fig. 8B). These results demonstrated that *AgMYB5* expression in transgenic *A. thaliana* significantly promoted antioxidant enzymatic activities and inhibited the accumulation of ROS induced by drought stress.

### Up-regulated drought-responsive genes of *AgMYB5 *transgenic *Arabidopsis*

**Fig. 9:**
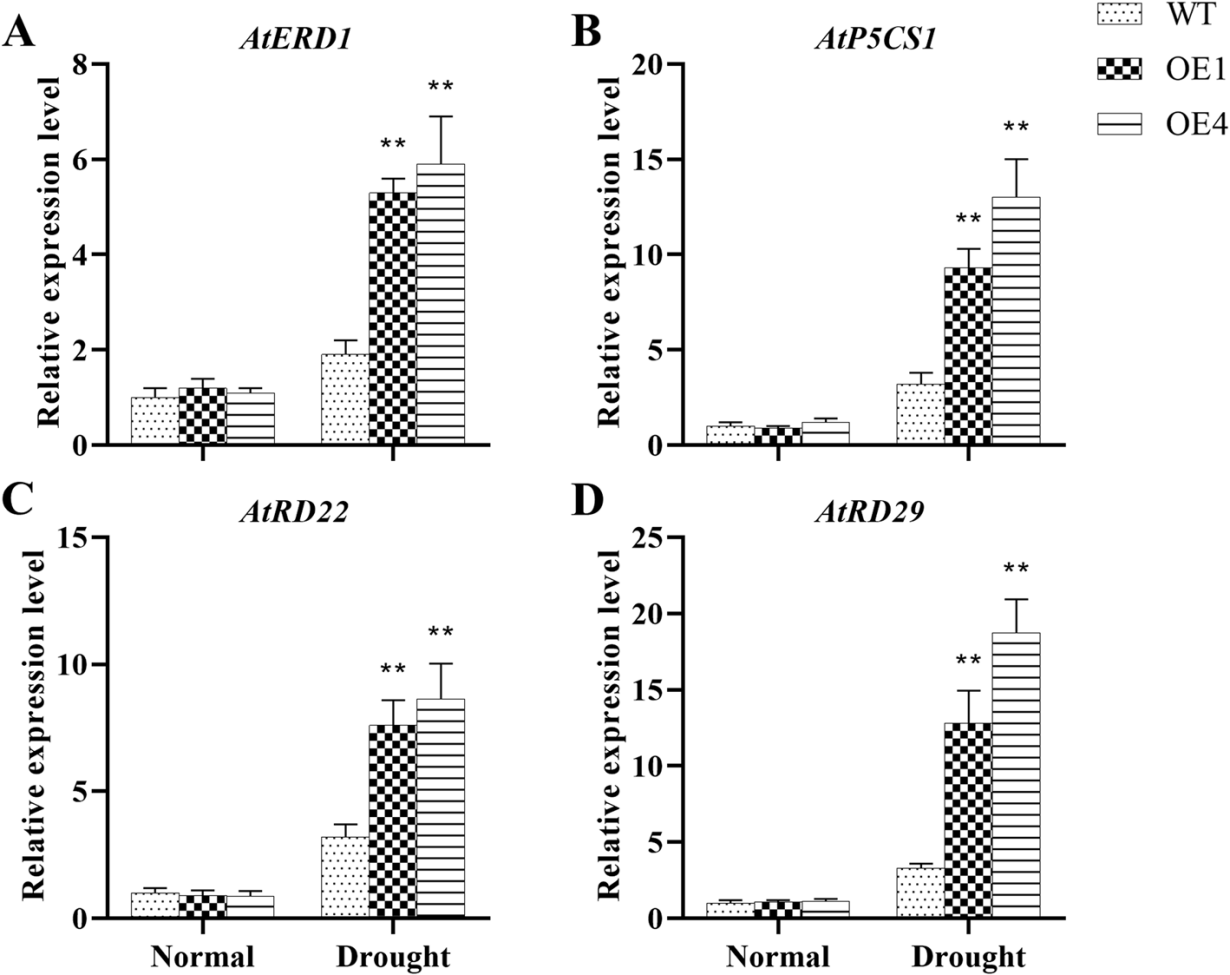
Relative expression levels of drought-response genes, including **(A)** *AtERD1*, **(B)** *AtP5CS1*, **(C)** *AtRD22*, and **(D)** *AtRD29*, in WT and transgenic lines (OE1 and OE4) under normal and drought conditions. The asterisk indicates a statistically significant difference (^*^, *P* < 0.05; ^**^, *P* < 0.01)

Four drought-responsive genes, including *AtERD1*, *AtP5CS1*, *AtRD22*, and *AtRD29*, were screened out to determine the effects of *AgMYB5* ectopic expression on transgenic *A. thaliana*. We observed relatively similar gene expression patterns in WT and transgenic *Arabidopsis* plants under normal conditions. Under drought conditions, relative expression levels of *AtERD1* (Fig. 9A), *AtP5CS1* (Fig. 9B), *AtRD22* (Fig. 9C), and *AtRD29* (Fig. 9D) were significantly higher in transgenic lines (especially OE4) than that in WT. The data of *AtERD1* was chosen as an example to illustrate the expression pattern. Under drought stress, compared to that of WT, the relative expression level of *AtERD1* increased by 1.79-fold (*P* < 0.0001) in OE1 and 2.11-fold (*P* < 0.0001) in OE4. These results indicated that *AgMYB5* enhanced drought tolerance of transgenic *Arabidopsis* by up-regulating the expression profiles of drought-response genes.

## Discussion

In plants, TFs usually exhibit differential expression patterns in different tissues and under various abiotic stresses. Kundan et al. identified 99 R2R3-MYBs from *Cannabis sativa*, and these TFs were found to have tissue-specific expression [46]. Here, we found that under various abiotic stress conditions, *AgMYB5* showed different expression patterns in celery, as well as tissue specificity. Moreover, under drought stress, *AgMYB5* expression was significantly higher in the root, which might be because when soils become dry, roots sense water scarcity earlier than the other tissues and continuously release root-source signals [47]. MYBs, MADSs, NACs, and WRKYs are responsible for carotenoid biosynthesis and degradation [48]. Among them, MYBs played a key role in regulating β-carotene production. In *Liriodendron chinense*, based on genome-wide association, HPLC, and qRT-PCR, four MYBs were identified to have the function of regulating β-carotene accumulation in petals [49]. In the green mutant of *Citrus reticulata*, *CrMYB68* suppressed α- and β-carotene accumulation by down-regulating *BCH2* and *NCED5* [50]. RNA-Seq analysis revealed that *SlMYB72* affected β-carotene synthesis by regulating the expression of *SlPDS*, *SlZDS*, and *SlLCYB*, while RNA interference with *SlMYB72* inhibited lycopene and β-carotene accumulation [51]. In the present study, it was found the changes in *AgMYB5* expression and β-carotene content were closely related in celery, and ectopic expression of *AgMYB5* in *A. thaliana* promoted β-carotene accumulation and up-regulated β-carotene synthesis-related genes in a water-deprived environment, which confirmed the involvement of *AgMYB5* in β-carotene biosynthesis under drought stress. At the same time, β-carotene, an excellent natural antioxidant, confers stress resistance to plants by scavenging excess ROS [52]. In *Dioscorea esculenta*, the over-production of β-carotene led to a stronger DPPH radical-scavenging capacity and a higher photosynthetic efficiency [53]. Our results showed that drought stress induced the activities of SOD, POD, and CAT, and increased the contents of MDA, H_2_O_2_, and O_2_^−^ at various levels. Moreover, compared with that in WT, increased antioxidant enzymatic activities and decreased ROS contents were found in transgenic lines, which indicated that *AgMYB5* expression protected ROS scavenging system by enhancing β-carotene production.

It has been reported that in the ABA biosynthesis of plants, NCED is the key enzyme, and β-carotene acts as the precursor [54, 55]. In transgenic *Oryza sativa*, *OsZDS* overexpression enhanced β-carotene biosynthesis, which also promoted *OsNCED1* expression and induced ABA accumulation [56]. In *A. thaliana*, ABA treatment induced *AtD27* (a gene encoding β-carotene isomerase) expression, and the *AtD27* mutant exhibited lower ABA content [57]. In the present study, compared to WT, ABA content and expression profiles of ABA biosynthesis-related genes were higher in transgenic lines, revealing that under drought stress, *AgMYB5* promoted endogenous ABA biosynthesis by enhancing β-carotene accumulation. In drought-stressed plants, ABA is a major phytohormone involved in regulating stomatal aperture [58]. According to the reports from *Populus euphratica* [59], *Oryza sativa* [60] and *Solanum lycopersicum* [61], plants increased drought tolerance by modulating stomatal closing. Our results demonstrated that compared to WT, *AgMYB5* inhibited water loss by regulating stomatal closure, thereby enhancing drought tolerance of transgenic *Arabidopsis*.

Recently studies have shown that TFs respond to biotic and abiotic stress by cooperating with stress-response genes. In *Gossypium hirsutum*, *GhWRKY17* overexpression significantly reduced drought and salt tolerance by down-regulating the transcript levels of *GhERD* and *GhLEA* [62]. Compared to WT, *VvNAC17* transgenic *Arabidopsis* showed stronger drought tolerance and enhanced expression of *AtCOR47*, *AtP5CS*, *AtRD22*, and *AtRD29A* [63]. Our study found that under drought stress, the expression of *AtERD1*, *AtP5CS1*, *AtRD22*, and *AtRD29* were significantly enhanced in *AgMYB5* transgenic lines as compared to that in WT, indicating that through activating the expression of drought stress-response genes, *AgMYB5* enhanced drought tolerance of transgenic *Arabidopsis*. Previous reports showed that *AcMYB3R*, obtained from *Actinidia chinensis*, activated the expression of *AtRD22*, *AtCOR15A*, and *AtRD29*, which led to enhanced drought tolerance of transgenic *Arabidopsis* [64]. A similar study also reported that overexpression of *TaSIM*, an R2R3-MYB gene of *Triticum aestivum*, boosted the expression of *AtRD22* and *AtRD29*, which revealed the function of *TaSIM* in the drought response mechanism [65].

**Fig. 10:**
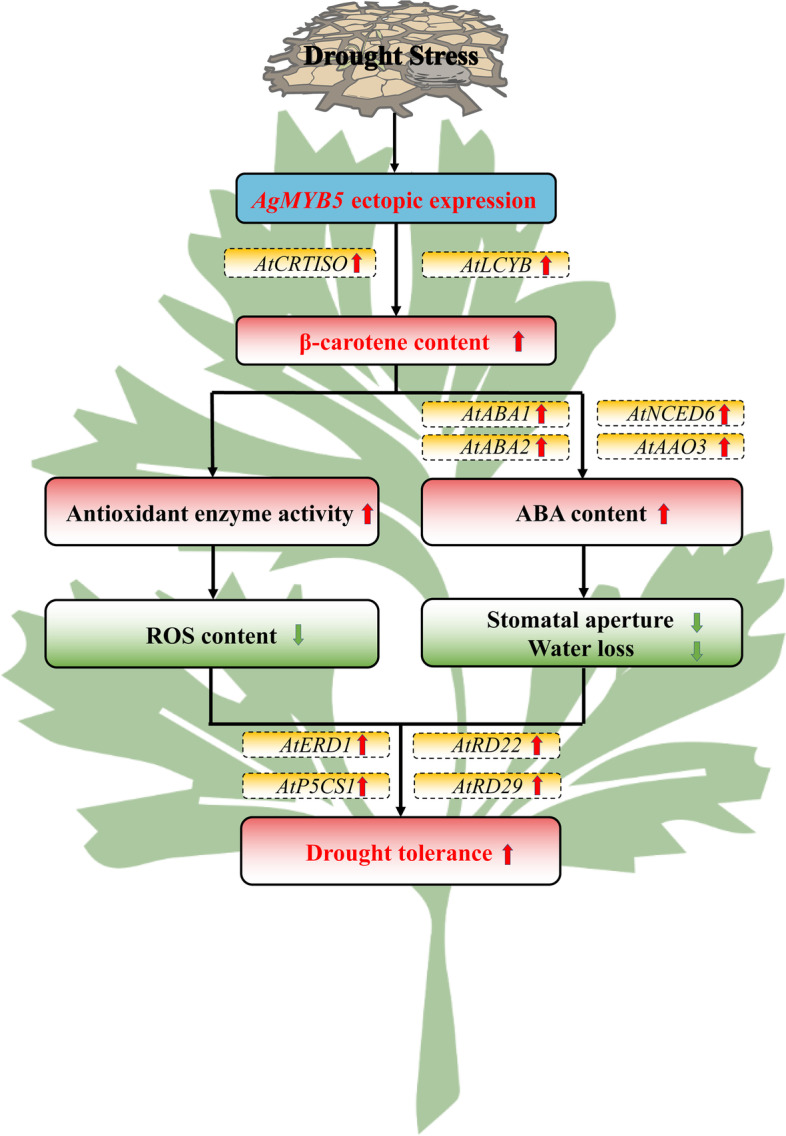
The potential regulation model of *AgMYB5* in β-carotene biosynthesis and drought tolerance of transgenic *Arabidopsis*

In conclusion, we isolated a transcription factor *AgMYB5* from the celery cultivar ‘Jinnanshiqin’. *AgMYB5* presented the properties of transcriptional auto-activation and tissue specificity under drought stress, as well as a correlation with carotenoid accumulation. In transgenic *Arabidopsis*, *AgMYB5* ectopic expression enhanced β-carotene biosynthesis and increased β-carotene content under drought stress, which in turn protected transgenic *Arabidopsis* against ROS-mediated peroxidation. Meanwhile, β-carotene accumulation induced ABA biosynthesis, which triggered stomatal closure and reduced water loss of transgenic *Arabidopsis* under drought stress. In addition, drought-response genes were significantly up-regulated by *AgMYB5* ectopic expression (Fig. 10). Interestingly, unlike that under normal conditions, β-carotene and ABA accumulation, antioxidant enzyme activity, and stomatal aperture in transgenic *Arabidopsis* showed significant differences under drought stress, implying that *AgMYB5* was barely expressed under normal conditions but induced by drought stress. Altogether, our findings suggest that *AgMYB5* promotes β-carotene accumulation and plays a pivotal role in the drought tolerance of *Arabidopsis*. However, the anti-drought ability validation of *AtMYB5* from *A. thaliana* is still required, which will give a more powerful reference for MYB5 function investigation. Meanwhile, *AtMYB5* function validation will be an excellent subject for us to study the molecular regulation mechanism of MYBs in plant drought resistance.

## Supplementary Information

**Additional file 1:** **Figure S1.** Copy number of *AgMYB5* in transgenic *Arabidopsis* lines (OE1 and OE4).

## Data Availability

*AgMYB5* sequence data from ‘Jinnanshiqin’ in this study has been submitted to the NCBI database with accession No. OP272489. Sequence data used in this article can be found in the GenBank database (http://www.ncbi.nlm.nih.gov/Genbank) under the following accession numbers: AtMYB5 (NP_187963.1); CsMYB5 (QSV39846.1); DlMYB5 (QRV61369.1); LcMYB5 (QXT50540.1) and NtMYB5 (XP_009601374.1). The data sets supporting the conclusions of this article are included within the article. Celery (*A. graveolens* L., cv. ‘Jinnanshiqin’) and *Arabidopsis thaliana* (‘Columbia-0’) were deposited at the State Key Laboratory of Crop Genetics and Germplasm Enhancement, Nanjing Agricultural University (32°04′N, 118°85′E).
