# Supplementary material for: AgMYB5, an MYB transcription factor from celery, enhanced β-carotene synthesis and promoted drought tolerance in transgenic Arabidopsis
Source: BMC Plant Biol. 2023 Mar 21;23:151. doi: 10.1186/s12870-023-04157-3 (PMC10029358; doi:10.1186/s12870-023-04157-3)
Supplement: Supplementary file 1 — Additional file 1: Figure S1. Copy number of AgMYB5 in transgenic Arabidopsis lines (OE1 and OE4). [file 12870_2023_4157_MOESM1_ESM.docx]

Figure S1. Copy number of *AgMYB5* in transgenic *Arabidopsis* lines (OE1 and OE4).
